# Clinical Challenges in the Diagnosis and Treatment of Temporal Bone Osteomyelitis

**DOI:** 10.1155/2017/4097973

**Published:** 2017-04-09

**Authors:** Liubov Kornilenko, Saulius Rocka, Svajunas Balseris, Irina Arechvo

**Affiliations:** ^1^Faculty of Medicine, Vilnius University, Ciurlionio 21/27, LT-03101 Vilnius, Lithuania; ^2^Clinic of Neurology and Neurosurgery, Faculty of Medicine, Vilnius University, Siltnamiu 29, LT-04130 Vilnius, Lithuania; ^3^Department of Ear, Nose and Throat Diseases, Republican Vilnius University Hospital, Siltnamiu 29, LT-04130 Vilnius, Lithuania

## Abstract

Temporal bone osteomyelitis is a serious life-threatening condition—a quick and proper diagnosis is needed to start treatment and reduce morbidity and mortality. Changing trends of the disease make a differential diagnosis difficult. To emphasize the importance of a clinical suspicion of this dangerous condition, our experience with three difficult cases is presented. The diagnosis was based on clinical symptoms, otoscopic findings, and findings on computed tomography or magnetic resonance imaging. Neoplasm and granulomatous inflammation were excluded by multiple biopsies. The disease can develop in nondiabetic patients. The disorder might be related to the initial inflammatory process in the middle ear with further direct spreading of infection through defects in the bony walls to deep temporal bone structures. Imaging should be performed early to detect osteolytic lesions of the skull base. Surgery was used for the presence of bone sequestra and infratemporal abscess.

## 1. Introduction

Temporal bone osteomyelitis (TBO) is a dangerous condition, and the proper treatment at the appropriate time is extremely important. Fatal cases of temporal bone osteomyelitis have been described previously. In the past, its cause was almost always necrotizing external otitis (NEO), especially in older adults with diabetes mellitus (DM) and patients with immunosuppressive conditions [1, 2]. The other rare etiologic conditions also mentioned in the literature are an infection of the middle ear or previous mastoidectomy [3–5]. Changing trends in the clinical picture of the disease, including a declining prevalence of DM and atypical causative agents, have been reported recently [6]. In the current manuscript, we present our experience with three diagnostically difficult cases of the disease to highlight this dangerous condition. We also noticed some new clinical details in the histories of our patients. The charts of the patients diagnosed with TBO and treated at the Department of Ear, Nose and Throat Diseases of Republican Vilnius University Hospital from December 2013 to August 2014 were retrospectively analyzed. The diagnosis was based on the following criteria: persistent otalgia and otorrhea; granulation tissue in the external auditory canal (EAC) and/or tympanic cavity; the tumor and specific inflammation were excluded by the multiple biopsies; typical findings on computed tomography (CT) or magnetic resonance imaging (MRI). Demography, bacteriology, comorbid diseases, and imaging data were systematically reviewed.

## 2. Case Report One

A 64-year-old man presented to the tertiary referral hospital with a 1-month history of severe otalgia, constant otorrhea, and a 1-week history of facial weakness on the left side. He also complained of painful mastication on the same side. He had no previous history of ear infection, diabetes mellitus, or tuberculosis. The patient had a history of excessive alcohol consumption.

At arrival, his temperature was 37.2°C. A blood test revealed leukocytosis (12.3 × 10^9^/L) and normal glucose level. On physical examination, the patient had a facial paralysis grade IV according to the House-Brackmann scale. Otoscopically, there was edema with granulation tissue in the EAC and an eardrum perforation in lower quadrants on the left side. Pus and granulations were also detected in the tympanic cavity on that side.

**Figure 1:**
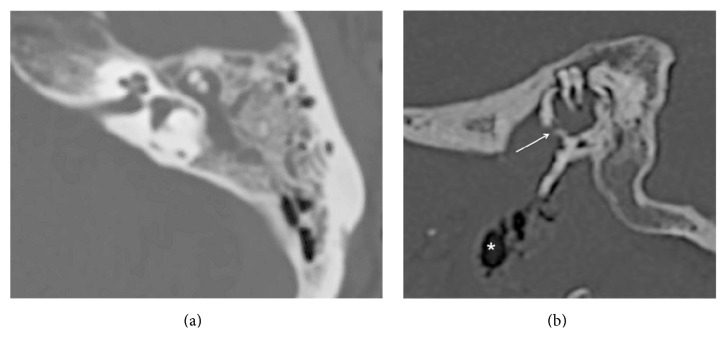
(a) Mastoiditis on the left side. There is diffuse debris throughout the mastoid. (b) An erosion of the anterior-inferior wall of the middle ear cavity (arrow) with air bubbles in the left prestyloid compartment (asterisk).

Radiologically, mastoiditis was diagnosed (Figure 1(a)). CT scans also showed erosion of the anterior-inferior walls of the middle ear cavity and external auditory canal (Figure 1(b)). Air bubbles in the prestyloid compartment and TMJ were also detected.

From the geniculate ganglion to the stylomastoid foramen, no obvious facial nerve bony canal destruction was detected. Multiple biopsies showed highly vascularized granulation tissue with leukocytic infiltration and foci of necrosis. They did not reveal any neoplasm or granulomatous inflammation. A culture showed a growth of *α-hemolytic Streptococci*.

**Figure 2:**
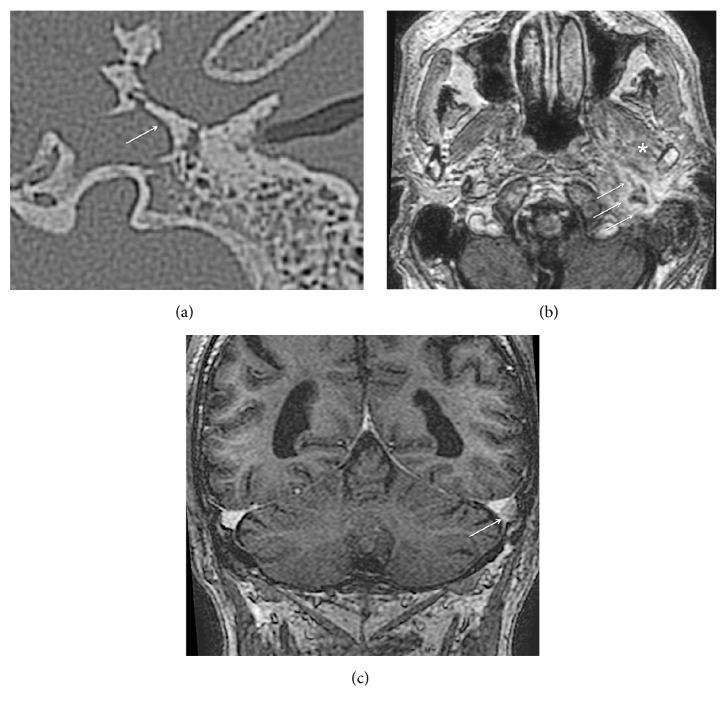
(a) CT scan of the left temporal bone—a bony sequestrum in the tympanic part is shown by the arrow. (b) An abscess in the left infratemporal fossa appears as a contrast-enhanced mass on the periphery, with irregular contours (arrows). A trismus was explained by inflammatory infiltration of the lateral pterygoid muscles (asterisk). (c) A thrombosed sigmoid sinus is seen on the left side (arrow).

Treatment with intravenous antibiotics (Cefuroxime 750 mg three times daily, Metronidazole 500 mg twice daily, and Ciprofloxacin 200 mg twice daily), aural suction, and topical Ciprofloxacin and Dexamethasone drops was started immediately. However, conservative management and intensive aural toilet were ineffective, and the patient underwent surgery. Canal wall down mastoidectomy was performed. Removal of the incus was required to reestablish the patency of the blocked aditus ad antrum and attic. A dehiscent facial nerve in the tympanic portion was observed intraoperatively. Ossiculoplasty with repositioning of the incus was performed at the same procedure. However, after a short improvement, purulent otorrhea and pain in the preauricular pain reoccurred. New massive granulations in the anterior part of the tympanic cavity were observed. Pus in the canal appeared with closure of the mouth and clenching. Repeated CT scan of the temporal bones and temporomandibular joint (TMJ) revealed a bony sequestrum and defect in the anterior middle ear cavity and external auditory canal walls (Figure 2(a)). MRI with intravenous contrast was carried out; in the left infratemporal fossa, an abscess (30 × 9 mm) enhanced on the periphery with the irregular contours was detected (Figure 2(b)). The abscess extended from the external auditory canal towards the jugular fossa, carotid, and parapharyngeal compartments. Debris in the mastoid on the left was described as well. Altered intensity of the lateral pterygoid muscle on the left as well as thrombosed internal jugular vein and partially thrombosed transversal and sigmoid sinuses were detected on the left (Figures 2(b) and 2(c)).

**Figure 3:**
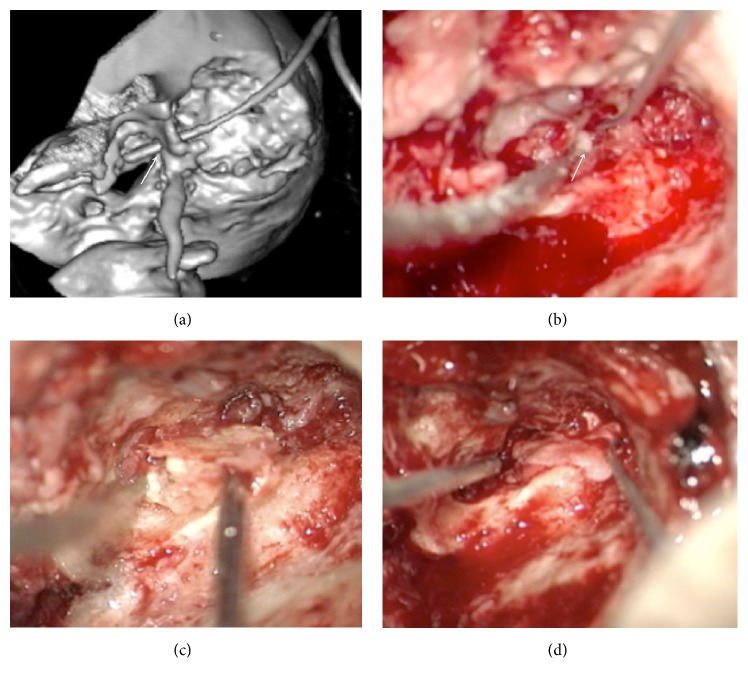
(a) A 3D volume rendering scan shows the amount of bone removed during lateral petrosectomy. The drain was inserted into the abscess cavity through the defect in the anterior EAC wall (arrow). (b) Intraoperative image: a defect in the anterior wall of the tympanic cavity (arrow). Pus under the pressure appeared when the granulations were removed from the tympanic cavity. (c) After the tympanic part of the temporal bone was partially drilled, a large bony sequestrum was removed with microforceps. (d) Finally, the third type of tympanoplasty was carried out.

A final clinical diagnosis of temporal bone osteomyelitis complicated by infratemporal abscess coexisting with mastoiditis and necrotizing external otitis was made on the basis of medical signs and imaging data. The second operation was carried out in collaboration with a neurosurgeon (S. R.). Lateral petrosectomy was performed, and a drain was inserted into the abscess cavity (Figure 3(a)). Granulations were removed from the middle ear cavity. A defect in the anterior wall was identified (Figure 3(b)). The tympanic part of the temporal bone was drilled out, and a large sequestrum was removed (Figure 3(c)). Next, tympanoplasty type III using temporal muscle fascia was performed (Figure 3(d)).

**Figure 4:**
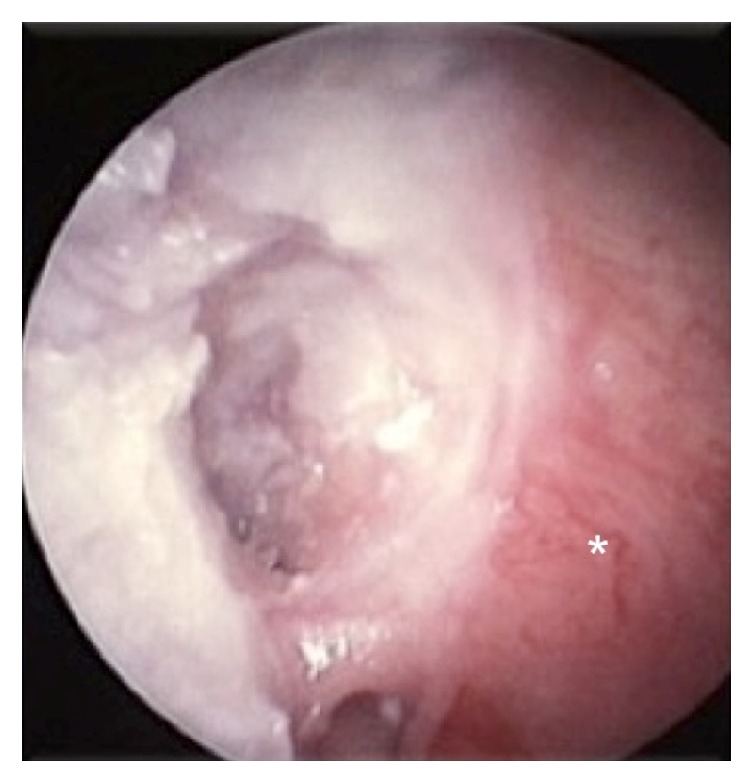
Six months postoperatively, a partially epithelized mastoid bowl (asterisk) with an intact tympanic membrane was seen.

The patient continued to use Ciprofloxacin 500 mg twice a day for six weeks after surgery. On follow-up, six months postoperatively, the tympanic membrane was intact, and the mastoid bowl was noncompletely epithelized (Figure 4).

Grade V House-Brackmann facial paresis persisted at the last follow-up.

## 3. Case Report Two

A 85-year-old man presented to the emergency department of our hospital with a 1-month history of severe otalgia and otorrhea on the right side. At arrival, the patient also complained of hoarseness and difficulties and pain when swallowing. He denied a history of DM. A craniotomy due to a hemorrhagic stroke was performed ten years ago. On physical examination, the VII, IX, and X cranial nerves were affected. Otoscopically, there were pus and granulations in the EAC and tympanic cavity. A blood test showed a moderate leukocytosis (15.8 × 10^9^/L). The histopathological examination revealed granulation tissue and purulent inflammation with massive neutrophilic infiltration.* P. aeruginosa* was isolated from the ear swab.

**Figure 5:**
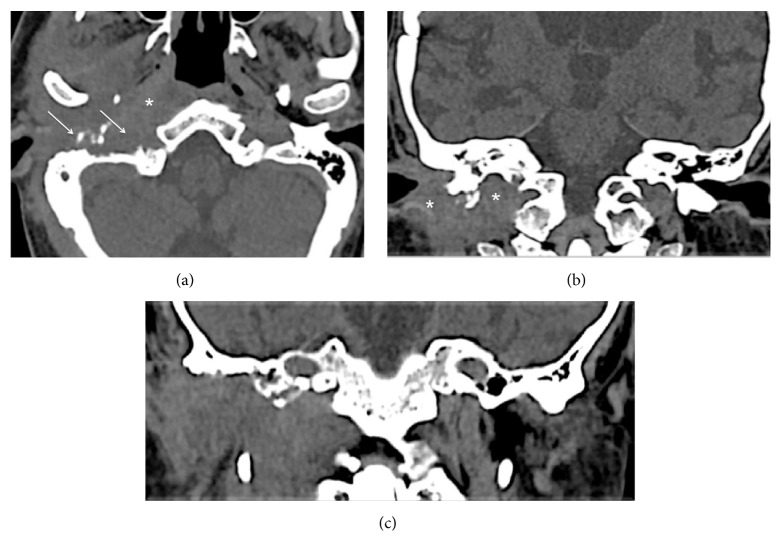
(a) Inflammatory infiltration at the level of the prevertebral and superior pharyngeal constrictor muscles (asterisk). Arrows show the massive erosion of the tympanic part of the temporal bone. (b) Inflammatory changes at the level of the jugular fossa and parotid compartment (asterisks). (c) A soft tissue lesion in the parapharyngeal and parotid spaces.

Initially, on the head CT scans, diminished aeration of the mastoid air cells and tympanic cavity without bone destruction was described. No fluid collection was observed on the neck CT scans. The diagnosis of acute complicated middle ear and external otitis was proposed in the emergency room, and the patient was admitted to the otorhinolaryngology department. Massive intravenous antibiotic therapy (Penicillin G 2 mln. three times daily, Gentamycin 240 mg daily, and Ceftazidime 1 g three times daily) with local debridement of the aural granulations was started. Feeding a patient through a nasogastric tube was required due to severe dysphagia. On the following day, the CT scans were reevaluated by an otologist (I. A.) and an experienced neuroradiologist. Advanced inflammatory infiltrate at the central skull base was observed that involved the parapharyngeal, retropharyngeal, carotid, parotid, retroparotid, and prevertebral compartments, as well as extending to the jugular foramen (Figures 5(a)–5(c)). The intensity of the prevertebral and superior pharyngeal constrictor muscles was pathologically changed.

**Figure 6:**
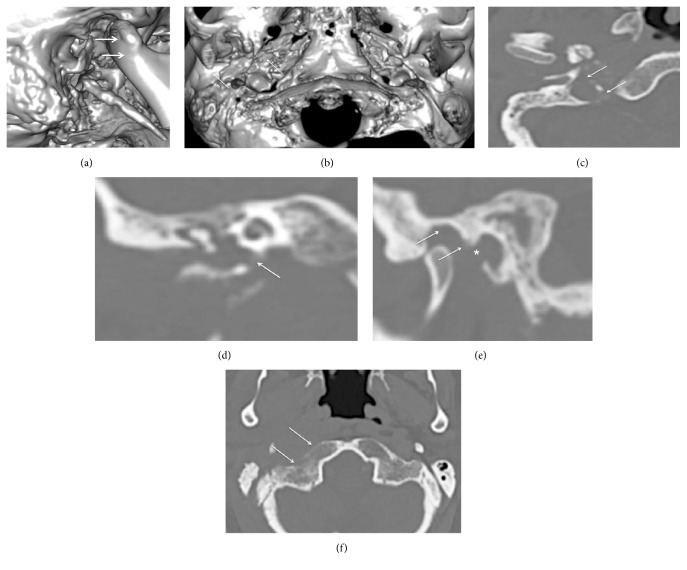
(a) The 3D volume rendering technique shows the lateral surface of the temporal bone: the destruction of the base of the styloid process (vaginal process) is clearly seen. Anterior dislocation of the mandible with subluxation of the condyle is shown with arrows. Note slight cortical erosion of the mastoid process of the right side. (b) Inferior view of the skull base: massive destruction of the petrous and tympanic parts of the temporal bone is marked with arrows. (c) The most severe bone lytic changes were observed around the carotid foramen (arrows). (d) A destruction of the carotid plate is clearly seen on this coronal reformat (arrow). (e) The other possible infection-spreading pathway was from the external auditory canal. Asterisk shows a defect in the anterior wall of the latter. Additionally, arrows show erosion of the mandibular fossa roof as well as auricular tubercle. Note an anterior dislocation of the mandibular condyle. (f) Axial CT scan shows the destruction of the cortical layer of the occipital bone (arrows).

The most severe bony destruction was observed in the temporal and occipital bones: squamous, petrous, and tympanic portions of the former were involved (Figures 6(a)–6(f)).

A neurosurgeon was consulted regarding the patient. The clinical diagnosis of the otogenic central and lateral skull base osteomyelitis with jugular foramen syndrome due to the acute middle and NEO was proposed. We chose conservative treatment while awaiting MRI. The latter was indicated to differentiate the disease from the neoplastic skull base lesions. Despite the intravenous antibiotic therapy, the general condition of the patient deteriorated, and he died suddenly in three days because of acute cardiovascular insufficiency. The relatives refused the autopsy.

## 4. Case Report Three

A 93-year-old man presented to the emergency department of our hospital with a 1.5-month history of severe otalgia and constant otorrhea on the right side. He had no previous history of otologic diseases, DM, or lung tuberculosis. Otoscopically, the bony part of the EAC was partly obstructed by massive fleshy granulations. Granulations were also observed in the tympanic cavity through the eardrum perforation. A blood test showed leukocytosis (18.2 × 10^9^/L).* S. aureus* was isolated from the ear swab.

**Figure 7:**
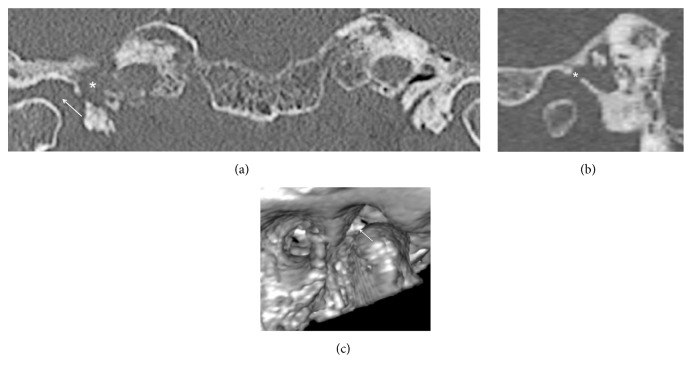
(a) The asterisk shows erosion of the temporal bone around the carotid canal (peritubal mastoiditis). The arrow shows a widened TMJ cavity. (b) Defect in the anterior tympanic cavity wall (asterisk). (c) A 3D volume rendering technique shows the same defect from the fossa mandibularis perspective (arrow).

CT scan revealed an osteolytic lesion of the petrous apex of the temporal bone with loss of clear bony margins of the carotid foramen (Figure 7(a)). Radiologically destructive peritubal mastoiditis was diagnosed. Several bony sequestra were clearly seen on the CT scans. In this case, an infection extended directly through the defect in the anterior wall of the tympanic cavity into the surrounding spaces: mandibular fossa and temporomandibular joint (Figures 7(b) and 7(c)). Diffuse pericapsular inflammation of the latter with widened joint cavity was seen on the CT scans.

Clinically, a diagnosis of coalescent mastoiditis complicated by temporal bone osteomyelitis coexisting with necrotizing external otitis was made.

Due to the age and anesthetic risk, a conservative tactic was chosen with active removal of the granulations under local anesthesia. After the treatment, subjective improvement of otalgia and reduction of the granulations were observed. The patient was discharged from the hospital after one week. Long-term antibiotics (Cefuroxime 500 mg twice a day for four weeks) with local drops were prescribed according to the culture results.

## 5. Discussion

Temporal bone osteomyelitis is a rare but very aggressive disease with different etiologies. To reduce morbidity and mortality, it should be recognized early, even in the emergency department, as the final outcome depends on early specific antibiotic therapy. The presented cases show that TBO can be local, or the disease can involve adjacent structures of the skull base.

According to the literature, the gold standard for the diagnosis and monitoring of osteomyelitis of the base of skull is radioisotope scanning [7]. However, these diagnostic tools are quite expensive and time consuming, and the high radiation can restrict their usage [8]. We have no opportunity to use scintigraphic imaging in our facility. In the present report, we showed that thorough analysis of the high-resolution CT and MRI findings with advanced postprocessing techniques (multiplanar reconstructions, 3D volume rendering) could exactly detect the erosive change in the temporal bone and skull base. Nevertheless, the disease remains a challenge because the first clinical symptoms may be atypical and subtle. In the emergency department, there is often no possibility of consulting with an experienced neuroradiologist and osteolytic changes (as in case of the second patient) can be missed. Later, the erosive changes in the different parts of the temporal bone, temporomandibular joint, and mandibular fossa were clearly delineated on CT scans. Our findings are in agreement with the results of previous investigators [9, 10]. However, a computed tomography is limited in use for monitoring the response to treatment because demineralization changes are detected even in the beginning of disease and persist despite resolution of the clinical symptoms [2, 11]. Previous authors have stated that CT and MRI are even more useful than nuclear medicine studies in the assessment of NEO [12] and temporal bone osteomyelitis. In the present study, an infratemporal abscess with the involvement of the muscles of mastication was diagnosed only with MRI. The initial CT scan revealed only the indirect signs of the abscess—air bubbles in the prestyloid compartment and large bony sequestrum of the tympanic part of the temporal bone. MRI also showed the exact position of the thrombus in the sigmoid dural sinus. It is present in 10–20% of TBO cases [13].

Often, the infection comes from the osseous-cartilaginous junction of the external auditory meatus [12, 14, 15]. In all of our cases, the defects in the anterior bony walls of the EAC and tympanic cavity were possible infection-spreading pathways. Facial neuropathy is the most common neuropathy observed in NEO [16]. We observed it in two of three cases. In the first case, the dehiscent facial nerve was damaged in its tympanic portion. The possibility of damage in the area of the stylomastoid foramen could not be excluded. In the second case, a large destructive cavity was observed in this area, indicating the nerve damage site. The spreading pathway of infection from the EAC to the skull base through the stylomastoid foramen was described by previous investigators [3, 17]. It was shown that involvement of the stylomastoid foramen will lead to facial paralysis in 25% of patients [18, 19]. Less frequently, jugular foramen nerves were affected—deficits in cranial nerves IX, X, and XI in that order occurs [16, 18, 19]. The second patient possessed a deficit of the multiple lower cranial nerves. In this case, the jugular foramen syndrome with possible lesions of the nerves in the retroparotid compartment was determined. Furthermore, jugular vein thrombosis may occur due to inflammation at this site [15]. We observed partial thrombosis of the sigmoid sinus in the first patient. Infection also may spread extracranially involving the infratemporal fossa, parotid, and neck, leading to the involvement of surrounding structures and formation of abscess [11].

The present report shows that the qualification of the radiologist as well as a high index of suspicion in case of otalgia, otorrhea, leukocytosis, and granulation tissue in the EAC is of great importance to evaluate the lesions of the temporal bone and skull base. It would be optimal that the experienced otologists, neurosurgeons, and radiologists participate in the analysis of the images.

It should be emphasized that, in the first and third cases, since the patients presented with widespread lesions and atypical causative agents, we were uncertain about the origin of the infection. Because all of our patients were treated with oral antibiotics without any clinical improvement before admission to the hospital, obtaining a positive culture might be expected to be difficult. However, in all of the presented cases, the cultures were positive:* alpha-hemolytic Streptococci*,* Pseudomonas aeruginosa,* and* Staphylococcus aureus* were the causes, respectively. Pseudomonal ear infections most often are the cause of skull base osteomyelitis, especially in diabetic or immunosuppressed patients [20, 21]. However, in the first patient, according to the literature, an atypical, very rare agent,* alpha-hemolytic Streptococcus*, was cultured. Recently, we described a case of local temporal bone osteomyelitis caused by granulomatous tuberculous infection [22]. Yeh et al. reported that in case of chronic otorrhea, otalgia, and granulation tissue in the ear a diagnosis of otomastoiditis with TBO caused by nontuberculous* Mycobacteria* should be suspected [23]. The authors concluded that acid-fast stain and mycobacterial culture should be performed in all suspicious cases. The current study was based on histological examination, which, according to Yeh et al., did not produce a highly sensitive diagnosis. Currently, a culture-based antibiotic therapy remains the main treatment option in skull base osteomyelitis.

As shown previously [24], NEO usually affects elderly patients with diabetes. The previous authors showed that up to 100% of the subjects were diabetic [25–27]. All three of our patients had a normal blood glucose level. However, two of them where over 85 years of age, and the immunosuppression condition and osteoporosis related to the advanced age could be the cause of the infection spreading fast from the external and middle ear. In the first younger patient (64 years old), a problem of alcoholism might have decreased the host defenses (deficiency in the immune system, malnutrition). Other conditions causing immunosuppression such as HIV/AIDS, chemotherapy-induced aplasia, refractory anemia, chronic leukemia, lymphoma, splenectomy, neoplasia, and renal transplantation, which could predispose patients to NEO according to the literature [16], were excluded in our patients. Our experience confirmed the data of recent research [7] that the patients without immunocompromise and/or diabetes mellitus can develop temporal bone osteomyelitis originating from NEO and middle ear infection. The known clinical conditions such as trauma, bone surgery, or other diseases that can affect the vascularity of bone (osteoporosis, osteopetrosis, Paget's disease, radiation, and malignancy) and predispose to TBO [28, 29] should be excluded anamnestically. In our oldest patient (93 years old), severe osteoporosis was noticed on the CT scans.

We used topical antibiotic drops in all cases, although, according to the literature, the use of topical antibiotics in NEO is controversial because of changing EAC bacterial flora and increasing antibiotic resistance without adding a significant benefit [2, 12, 16]. A more aggressive surgical technique should be used in the case of otitis media and necrotizing external otitis to remove all of the granulations from the tympanic cavity and external auditory canal. In the first case, the first operation was unsuccessful. Later, the infratemporal abscess was drained through the posterior approach when part of the tympanic bone was drilled first and the bony sequestrum was removed. Before the antibiotics era, NEO often caused death, with mortality rates of nearly 50% with surgical treatment [11, 16]. Currently, surgery in NEO cases is used only for biopsies to exclude malignancy, local debridement of granulation tissue, and bony sequestra or drainage of associated abscesses. Previously, Sreepada and Kwartler proposed decompression of the cranial nerves with rehabilitation of persistent cranial nerve neuropathy in highly selected cases [17]. In two of our cases, removal of the granulations from the external and middle ear improved the otological status and diminished otalgia. The surgical treatment of TBO is controversial because there is an opinion that surgical intervention may induce pathogens into the uninfected regions, leading infectious foci to spread widely via vascular and fascial planes [30].
